# Passive Beamforming Design of IRS-Assisted MIMO Systems Based on Deep Learning

**DOI:** 10.3390/s23167164

**Published:** 2023-08-14

**Authors:** Hui Zhang, Qiming Jia, Meikun Li, Jingjing Wang, Yuxin Song

**Affiliations:** Tianjin Key Laboratory of Optoelectronic Sensor and Sensing Network Technology, Nankai University, Tianjin 300350, China

**Keywords:** intelligent reflecting surface, passive beamforming, attention mechanism, unsupervised learning

## Abstract

In the intelligent reflecting surface (IRS)-assisted MIMO systems, optimizing the passive beamforming of the IRS to maximize spectral efficiency is crucial. However, due to the unit-modulus constraint of the IRS, the design of an optimal passive beamforming solution becomes a challenging task. The feature input of existing schemes often neglects to exploit channel state information (CSI), and all input data are treated equally in the network, which cannot effectively pay attention to the key information and features in the input. Also, these schemes usually have high complexity and computational cost. To address these issues, an effective three-channel data input structure is utilized, and an attention mechanism-assisted unsupervised learning scheme is proposed on this basis, which can better exploit CSI. It can also better exploit CSI by increasing the weight of key information in the input data to enhance the expression and generalization ability of the network. The simulation results show that compared with the existing schemes, the proposed scheme can effectively improve the spectrum efficiency, reduce the computational complexity, and converge quickly.

## 1. Introduction

With the basic standardization and commercialization of the fifth-generation (5G) wireless communication technology, both academia and industry are now focusing on the sixth generation of wireless technology. According to the Cisco Annual Internet Report (2018–2023) [1], it is projected that, by 2023, the total number of mobile users will increase from 5.1 billion in 2018 to 5.7 billion in 2023. However, the proliferation of interconnected devices and limited bandwidth resources has brought numerous challenges to high-speed communication.

In order to manage the growing number of users and bandwidth demands, future sixth-generation wireless communication networks are increasingly demanding in terms of data transmission rates, spectrum efficiency, energy efficiency, and ubiquitous connectivity. Some novel technologies have been proposed, such as multi-connectivity [2], next-generation optical access networks [3], terahertz communication, intelligent reflecting surface (IRS), etc. [4]. Among them, the IRS technology is considered one of the promising and efficient solutions [5]. Specifically, the IRS is a planar metamaterial surface equipped with a large number of passive reflecting elements connected to an intelligent controller, which can generate independent phase shifts or amplitude attenuations for incoming signals at each reflecting element in real time [5]. By properly designing its reflection coefficients, the reflected signals from the IRS can add up or cancel out signals from other paths, which can increase the received signal strength or mitigate co-channel interference, thereby enhancing the quality of the communication link between transmitter and receiver. The theoretical analysis showed that an IRS with *N* reflecting elements can achieve a total beamforming gain of $N^{2}$ [6]. In addition, despite the fact that smart antennas are capable of seamlessly integrating beamforming signals with sidelobe signals and adaptively forming optimal array directional beams to enhance system performance, as the radio frequency increases, electromagnetic waves are more susceptible to obstruction by objects, such as buildings in urban areas. Deploying additional relays and base stations to provide better network coverage consumes more resources. From an energy consumption perspective, what makes IRS attractive is the possibility of amplifying and forwarding the incoming signal without employing any power amplifier but rather by suitably designing the phase shifts. Therefore, compared to using smart antennas or conventional Amplify-and-Forward (AF) relays, an IRS consumes less power. Furthermore, the structure of an IRS can be easily integrated into the communication environment. As a result, it is expected that an IRS will outperform other related technologies, such as relays, backscatter, and active ground-based systems based on massive multiple-input–multiple-output (MIMO) [7]. Currently, the IRS has been widely applied in communication, such as improving network coverage [8], enhancing wireless spectrum efficiency [9], reducing data transmission power consumption [10], enhancing air computing performance [11], and achieving secure wireless communication [12].

The IRS can provide an indirect reflection path between base stations and users to enhance link quality and avoid communication blockage, thus requiring careful design of the passive beamforming of the IRS. In other words, the IRS-assisted communication system benefits from the passive beamforming gain to maximize the received signal at the receiver [6]. In IRS-assisted communication, the design of the optimal IRS phase configuration is crucial for enhancing system performance. Due to the unit-modulus constraint of the IRS phase shift and the large number of elements to be optimized, the entire optimization problem is inherently non-convex, making it difficult to achieve the optimal solution for the IRS phase configuration. Currently, many works were involved in how to optimize the IRS reflection coefficients. For example, an optimization algorithm based on fractional programming, gradient descent, and alternating maximization was proposed to jointly optimize the IRS phase-shift matrix and the corresponding transmit power allocation [10]. The alternating optimization (AO) and semi-definite relaxation (SDR) methods were used to optimize the transmission beam and the IRS phase-shift matrix for maximizing the achievable rate for their proposed IRS architecture with non-diagonal phase shifts [13]. Similarly, the SDR technique was used to optimize the IRS configuration to solve the weighted sum-rate maximization problem [14]. In [15], a single-input–single-output (SISO) scenario was considered, and the successive convex approximation (SCA) and AO algorithms were used to improve the system’s spectral efficiency (SE). The achievable rate was maximized by combining alternating maximization with optimization minimization in a multi-user scenario [16]. Subsequently, a closed-form analytical solution was derived in [17] by imposing a rank-one constraint on the channel between the base station and the IRS. An AO method was proposed in [18] to solve the SE maximization problem in an IRS-assisted MIMO system. The fixed-point iteration and manifold methods were performed to optimize the active beamforming matrix at the base station and the phase of the IRS with the aim of maximizing the received signal in a multiple-input–single-output (MISO) system [19]. The iterative algorithm for maximizing the total received signal power through joint design of the transmit precoder and passive IRS phase shift was discussed [20]. The Lagrangian manifold and Riemannian manifold techniques were used to solve for the base station precoding transmit matrix and the IRS phase shift in a multi-IRS-assisted system [21]. The related work is summarized in Table 1. However, due to the iterative nature of these algorithms, their computational complexity was still high, which can lead to significant delays in real-time communication scenarios.

In recent years, deep learning has shown significant potential in handling non-convex optimization problems. In the context of IRS-assisted communication systems, deep learning methods have been employed to address non-convex optimization problems, including supervised learning [22], unsupervised learning [23,24], and deep reinforcement learning [25,26]. In the field of IRS-assisted wireless communication, deep learning demonstrated great advantages and potential in dealing with non-convex and high-dimensional optimization problems. For example, a novel two-stage neural network was proposed to solve the active and passive beamforming joint design problem in a multi-user MISO downlink system [24]. A multi-layer perceptron-based passive beamforming design was proposed [27], but it was only applied to single-antenna users. A novel active IRS architecture was proposed, and a deep neural network (DNN) model based on supervised learning was presented, which was trained offline by taking channel information from the active IRS as input to predict the IRS reflection beamforming [28]. Furthermore, a DNN model was trained by using received pilots as input instead of channel state information (CSI) to predict the optimal phase shifts of the IRS and beamforming vectors of the base station [29]. A DNN was designed for online configuration by mapping between user location data and optimal IRS phase shifts to maximize the received signal strength [30]. A deep reinforcement learning training model based on quantile regression was proposed to predict the reflection beamforming to optimize throughput in imperfect CSI scenarios [31]. The method in [32] focused on wireless secure communication systems assisted by an IRS, improving the system secrecy rate by jointly optimizing the beamforming of the base station and IRS under different users’ quality of service requirements and time-varying channels. A solution based on actor–critic deep reinforcement learning was proposed to optimize the base station beamforming transmit matrix and IRS phase-shift configuration in a MISO scenario [33]. Federated learning was used to increase the user data rate and protect the privacy data in IRS-assisted communication, which was applicable to a single antenna [34].

Through the analysis of existing optimization algorithms, it can be found that the practical applicability is usually limited due to their high computational complexity. In addition, in existing works using deep learning to solve non-convex optimization problems in an IRS, there is a general lack of exploration of input data and limited research on the structure of neural networks. The neural networks usually treat all input data equally, which leads to a lack of attention to key information in the data. Moreover, in supervised learning, the process of obtaining labels usually requires a significant cost, which is another challenge. Therefore, in this paper, we consider a more general downlink transmission system for IRS-assisted MIMO communication. We aim to optimize the IRS phase shifts to maximize SE. Specifically, the main contributions of this paper are given as follows:

- We propose an effective real-valued data input structure in which the indirect cascaded channel assisted by an IRS and the direct channel between the base station and the user are used as inputs. Unlike the existing input data structure [35], we improve it by extracting the real part, imaginary part, and absolute-value part as the real-valued input of the neural network. We use a three-dimensional (3D) data input structure and add an additional absolute-value part according to the problem’s characteristics.
- We propose an unsupervised learning scheme using a convolutional neural network (CNN) with an attention mechanism. The CNN has stronger feature extraction capability for data, while the attention mechanisms can help neural networks adaptively focus on key information and features in input data, increase the weight of important features, and better learn input data, thereby improving model computational efficiency. In addition, an additional penalty term is added to the loss function to ensure that the output satisfies the constraints, and unsupervised learning is used to save the cost of labels.
- The simulation results show that the proposed algorithm has good convergence and robustness. Compared with traditional optimization algorithms, the proposed algorithm greatly reduces the computational complexity while providing similar SE, demonstrating its potential and advantages in solving such problems.

The rest of this paper is organized as follows: The system model and the optimization problem formulation are described in Section 2. The specific model of the unsupervised learning scheme with an attention mechanism is presented in Section 3, including the input data, network structure, and loss function. Then, the simulation results are provided in Section 4. Finally, the conclusion is made in Section 5.

Notation: In this paper, we use bold-faced letters to represent vectors or matrices. For any matrix X, $X^{H}$ and $X^{-1}$ denote the conjugate transpose and inverse of the matrix, respectively. Log2(.) represents the logarithmic function, diag(x) represents the diagonal matrix composed of the vector x, the (k×k) identity matrix is denoted as $I_{k}$, and the imaginary unit of complex numbers is denoted as $j=\sqrt{-1}$. The Landau symbol O(.) denotes the complexity order.

## 2. System Model

**Figure 1:**
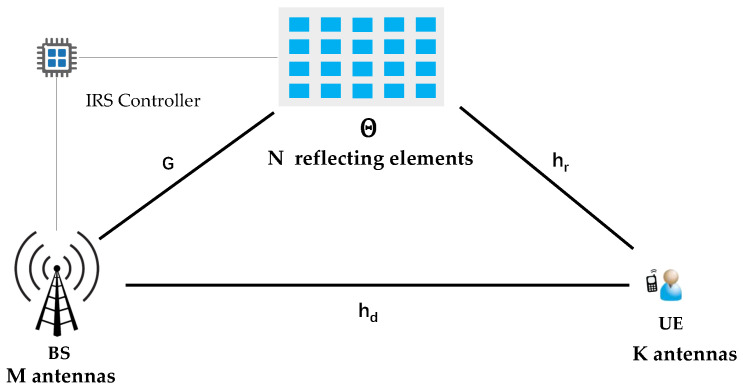
System model.

Consider a MIMO downlink transmission system composed of a base station with *M* antennas and a user with *K* antennas, as shown in Figure 1. In order to further improve the system performance, an IRS with *N* reflecting elements is used to assist the communication between the base station and the user. Furthermore, due to high path loss, signals reflected twice or more by the IRS are negligible. In particular, it is assumed that the CSI is known, and in practice, the CSI can be estimated via the methods in [36], but the consideration of imperfect CSI is left for our future work. Let $h_{d}\in C^{K\times M}$ denote the direct channel from the base station to the UE, while $G\in C^{N\times M}$ and $h_{r}\in C^{K\times N}$ are the indirect channels from the BS to the IRS, and from the IRS to the UE, respectively. The IRS adjusts the phase angles of its reflecting elements to maximize the user’s combined incident signal.

In addition, we define the IRS phase-shift vector as $\theta =\left[\theta_{1},\cdots ,\theta_{N}\right]$, and then its diagonal reflection phase-shift matrix is expressed as $\Theta =\mathrm{diag}\left(\left[\beta_{1}e^{j\theta_{1}},\cdots ,\beta_{N}e^{j\theta_{N}}\right]\right)\in C^{N\times N}$, where $0\leq \theta_{n}\leq 2\pi ,n=1,\cdots ,N$, $\theta_{n}$ is the reflection phase shift of the IRS, and $\beta_{n}\in \left[0,1\right]$ is its amplitude. The design reflecting elements of the IRS is to maximize the reflection signal, so β is usually set to 1 to obtain the maximum reflection gain, that is, the modulus of each reflection element is 1. Furthermore, we define the transmit signal vector as $x\in C^{M\times 1}$ and satisfy the transmit power constraint $E\left\{xx^{H}\right\}=P_{BS}I_{M}$, where $P_{BS}$ is the transmit power of the base station. The user receives the signal through an indirect channel assisted by the IRS and direct channel. Therefore, the received signal at the user can be expressed as

(1) $$\begin{array}{c} y=h_{r}\Theta Gx+h_{d}x+n=\mathrm{Hx}+n \end{array}$$

where $H\overset{\Delta }{\;=}h_{r}\Theta G+h_{d}$ is defined as the combining equivalent cascaded channels, $n∼CN(0,\sigma^{2}I_{K})$ represents the additive Gaussian white noise (AWGN) at the user, and $\sigma^{2}$ is the noise power. Based on Equation (1), the SE of the IRS-assisted MIMO system can be expressed as [18]

(2) $$\begin{array}{c} \mathrm{SE}\left(\left\{\theta_{n}\right\}\right)=\log_{2}\det\left(I_{K}+\rho HH^{H}\right) \end{array}$$

where $\left\{\theta_{n}\right\}=\left\{\theta_{1},\theta_{2},\cdots \theta_{N}\right\}$ is the IRS reflection phase-shift set that needs to be optimized, and $\rho =P_{BS}/\sigma^{2}$. Then, the passive beamforming design that maximizes the SE can be expressed as

(3) $$\begin{array}{cc} \left(P1\right)\; & \max_{\Theta }\;\log_{2}\det\left(I_{K}+\rho HH^{H}\right) \end{array}$$

(4) $$\begin{array}{cc} \; & s.t.\;0\leq \theta_{n}\leq 2\pi ,\forall n=1,2,\ldots ,N \end{array}$$

where Equation (4) is the unit-modulus constraint of the IRS phase shift, the objective function $\mathrm{SE}\left(\left\{\theta_{n}\right\}\right)$ is non-convex relative to $\left\{\theta_{n}\right\}$, and the feasible set of (P1) is non-convex, so (P1) is hard to find the optimal solution. The traditional iterative methods for solving such non-convex optimization problems include the AO algorithm [18], but it typically has high computational complexity. To reduce the computational complexity, a deep learning method assisted with an attention mechanism based on a CNN to solve non-convex optimization problems with the objective of maximizing SE is proposed. Specifically, we make use of a CNN to learn the mapping relationship between inputs and outputs and use attention mechanisms to enhance the expressiveness and generalization ability of the network.

## 3. Algorithm Design

We exploit an efficient input structure to improve model efficiency and propose a deep learning method based on unsupervised learning to solve the problem (P1), which is designed as a 3D CNN structure and integrated into the attention mechanism from the visual domain. The input of the model is the preprocessed CSI, the output is the optimal IRS configuration, and the output satisfies the constraints by designing a loss function.

### 3.1. Feature Design

Assume that $\bar{h}$ is a vector containing CSI parameters, which includes the IRS reflection channel G and $h_{r}$, as well as the direct channel $h_{d}$, i.e., $\bar{h}=\left\{G,h_{r},h_{d}\right\}$, so the phase shift learned by the CNN can be modeled as

(5) $$\begin{array}{c} \left\{{\hat{\theta }}_{n}\right\}=f_{\mathrm{CNN}}\left(\bar{h}\right) \end{array}$$

where $f_{\mathrm{CNN}}\left(\cdot \right)$ represents nonlinear mapping from $\bar{h}$ to $\left\{{\hat{\theta }}_{n}\right\}$ learned by the CNN. $\left\{{\hat{\theta }}_{n}\right\}$ represents the set of IRS phase shifts that need to be optimized. Considering that the efficiency and structure of the neural network depend largely on the input, designing $\bar{h}$ reasonably is the first and most important task of neural network modeling.

To design a more efficient input structure for $f_{\mathrm{CNN}}\left(\cdot \right)$, we first extract each individual phase shift $\theta_{n}$ from $\mathrm{SE}\left(\left\{\theta_{n}\right\}\right)$. Because the relationship about the phase $\left\{{\hat{\theta }}_{n}\right\}$ to be optimized in the objective function Equation (3) is implicit, we rewrite Equation (3) as an explicit function of $\left\{\theta_{n}\right\}$. We re-express the indirect channel G and $h_{r}$ as $h_{r}=\left[r_{1},\ldots ,r_{N}\right]$, $G={\left[g_{1},\ldots ,g_{N}\right]}^{H}$, where $r_{n}\in {}^{k\times 1}$ represents the *n*-th column of $h_{r}$, and ${g_{n}}^{H}\in {}^{1\times M}$ represents the *n*-th row of G. In addition, because Θ is a diagonal matrix, the combining equivalent cascaded channels can therefore be rewritten as

(6) $$\begin{array}{c} H^{\prime }=\sum_{n=1}^{N}e^{j\theta_{n}}r_{n}g_{n}^{H}+H_{d}=\sum_{n=0}^{N}e^{j\theta_{n}}H_{{}_{n}} \end{array}$$

where $H_{n}\overset{\Delta }{\;=}r_{n}t_{n}^{H}$, $H_{0}=H_{d}$. Furthermore, the optimization problem about the IRS phase can be rewritten as an explicit function about the IRS phase shift. For each $\theta_{n}$, there is the following expression:

(7) $$\begin{array}{c} \mathrm{SE}\left(\theta_{n}\right)=\log_{2}\det\left(A_{n}+e^{j\theta_{n}}B_{n}+e^{-j\theta_{n}}B_{n}^{H}\right) \end{array}$$

where $A_{n}$ and $B_{n}$ satisfy the following relationships:

$$\begin{array}{c} A_{n}\overset{\Delta }{\;=}I_{K}+\rho \left(\sum_{i=1,i\neq n}^{N}e^{j\theta_{i}}H_{i}\right){\left(\sum_{i=1,i\neq n}^{N}e^{j\theta_{i}}H_{i}\right)}^{H}+\rho H_{n}H_{n}^{H} \end{array}$$

(8) $$\begin{array}{c} B_{n}\overset{\Delta }{\;=}\rho H_{n}{\left(\sum_{i=1,i\neq n}^{N}e^{j\theta_{i}}H_{i}\right)}^{H} \end{array}$$

It can be observed that $A_{n}$ and $B_{n}$ are independent of $\theta_{n}$, and $A_{n}$ is full rank, so $A_{n}$ is an invertible matrix, and the objective function can be rewritten as:

$$\begin{array}{c} \mathrm{SE}\left(\left\{\theta_{n}\right\}\right)=\log_{2}\det\left(I_{K}+e^{j\theta_{n}}A_{n}^{-1}B_{n}+e^{-j\theta_{n}}A_{n}^{-1}B_{n}^{H}\right)+\log_{2}\det\left(A_{n}\right) \end{array}$$

This shows that if all variables ${\left\{\theta_{i}\right\}}_{i=1,i\neq n}^{N}$ are fixed, then $A_{n}$ and $B_{n}$ are both fixed, and then the objective function can be equivalent to maximizing Equation (9):

(9) $$\begin{array}{c} \log_{2}\det\left(I_{K}+e^{j\theta_{n}}A_{n}^{-1}B_{n}+e^{-j\theta_{n}}A_{n}^{-1}B_{n}^{H}\right) \end{array}$$

In particular, $\theta_{n}$ can be determined by Equation (10) [18]:

(10) $$\begin{array}{c} \theta_{n}=e^{-j\arg\left\{\lambda_{n}\right\}} \end{array}$$

where $\lambda_{n}$ represents the non-zero unique eigenvalue of $A_{n}^{-1}B_{n}$. This shows that the optimal $\theta_{n}$ required by the optimization problem can be obtained by $A_{n}^{-1}B_{n}$, and also, it can be calculated by $H_{n}$. Therefore, by using $\left\{H_{n}\right\}$ as the input of the neural network, the performance and efficiency of the model can be greatly improved, because the input can well reflect the role of each phase shift in the IRS reflection channel, allowing the neural network to more fully utilize the information provided by the data. Furthermore, obtaining $\left\{H_{n}\right\}$ only requires low-complexity matrix–vector multiplication. However, this input structure has a significant impact on the learning ability and structure of a CNN.

### 3.2. Data Preprocessing

For a two-dimensional (2D) CNN, its input data must be the value of the 2D real-number field; using complex numbers as inputs to neural networks may make it difficult to extract features [37], so $\left\{H_{n}\right\}$ needs to be preprocessed to obtain the 2D real-number matrix form required by the neural network. The existing splitting method commonly used for complex values is to split them into real and imaginary parts as two real-valued channels of the input data of the neural network. In contrast to directly serializing the CSI into a one-dimensional vector as the network input in [35], we adopt the approach in [38] of using three-channel data and processing it to construct three-channel two-dimensional input data. Compared with two-channel data, three-channel data contain more information than two-channel data, enabling the model to better learn the features of the input data. It also helps the model learn more generalized feature representations, facilitating the model’s application in diverse scenarios and thereby improving its performance and generalization capabilities [38]. Specifically, the data are first constructed as a 2D complex matrix, and then the real part and the imaginary part are split, and the absolute-value part is additionally added to form three-channel data as the input of the neural network.

**Figure 2:**
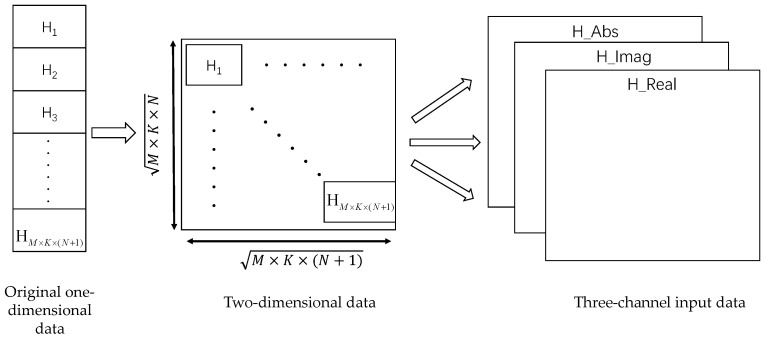
Three-channel data processing process.

It can be seen from the derivation that $\left\{H_{n}\right\}$ comprises (N+1) two-dimensional matrices of size M×K, so we first flatten it into a one-dimensional vector of length M×K×(N+1), and then convert it into a 2D complex square matrix of order $\sqrt{M\times K\times (N+1)}$. If $\sqrt{M\times K\times (N+1)}$ is not an integer, it is converted into a two-dimensional matrix of $\left(M\times K\right)\times \left(N+1\right)$ dimensions. After that, we take the real part, imaginary part, and absolute value of the 2D complex matrix, respectively, and transform it into three-channel real-valued data, as shown in Figure 2.

After performing the above data preprocessing process on $\left\{H_{n}\right\}$, we finally obtain the input data $X_{in}$, and its size is $\sqrt{M\times K\times (N+1)}\times \sqrt{M\times K\times (N+1)}\times 3$, where 3 represents the number of input channels of data.

### 3.3. Network Structure

In the single-antenna scenario, a DNN can be used to solve the optimal value [17]. However, when the number of antennas at the receiving end is greater than 1, it is more suitable to use a 2D CNN based on the feature dimension. Therefore, we propose a multi-layer CNN-based attention mechanism-assisted model to solve the phase-shift optimization problem of the IRS, which has excellent feature learning ability and robustness.

**Figure 3:**
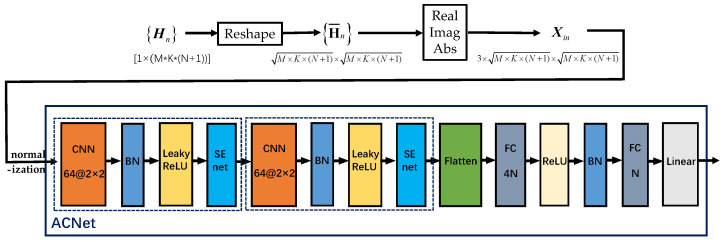
Data preprocessing and ACNet network structure.

**Figure 4:**
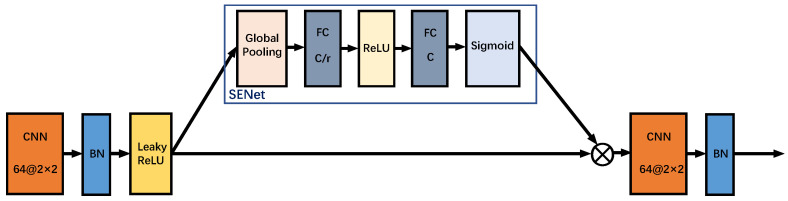
SENet structure.

As shown in Figure 3, the proposed network structure is denoted as attention-aided convolution net (ACNet). As shown in the diagram, the three-channel input data are obtained after data preprocessing. Before feeding it into the network, the input data are first normalized and then undergo feature extraction through two convolution–attention blocks. We design each convolutional layer with 64 filters of size 2 × 2. Specifically, the convolution operation slides the filters over the input feature matrix with a certain stride, resulting in an output feature matrix that serves as the input for the next layer. As indicated by the dashed boxes in Figure 3, a batch normalization (BN) layer is inserted after each convolutional layer to prevent overfitting and improve the model’s generalization ability [39]. Next, the leaky rectified linear unit (Leaky ReLU) activation function is applied to obtain nonlinear outputs, followed by a connection to the attention block, referred to as SENet [40], whose structure is depicted in Figure 4. After passing through two convolution–attention blocks, a flatten layer is connected to input the data into fully connected (FC) layers. Then, two FC layers are employed, with the sizes set as 4N and *N*, respectively. A rectified linear unit (ReLU) activation function is applied after the first FC layer, and a linear activation function is applied after the second FC layer. According to the approach in [41], each output of the network is processed according to Euler’s formula $e^{j\theta }=\cos\theta +j\sin\theta$ to better calculate the loss. The model’s weights are updated by computing the loss.

In order to effectively utilize the distribution characteristics between data channels, an attention mechanism is applied in the network structure design. In a traditional CNN, all features are given with the same importance for all data samples. However, for some data samples in practical applications, some features are more informative or important than others. Therefore, network performance can be improved by giving more weight to more informative features, i.e., feature importance reweighting. The attention mechanism can help the network focus on important features and ignore irrelevant or disturbing features.

We adopt the attention mechanism of SENet, and its structure is shown in Figure 4. In the SENet structure, it consists of an average pooling layer and two fully connected layers, and the sizes of the two fully connected layers are C/r and *C*, where *C* is the number of channels and *r* is the hyperparameter dimensionality reduction coefficient. In the SENet module, the features of each channel are averagely pooled through the average pooling layer first, and then the results are sent to two fully connected layers for feature importance weighting. The activation function after the fully connected layer uses ReLU and Sigmoid, where ReLU is used to extract the nonlinear part of the feature and Sigmoid is used to calculate the feature weight. Through the attention module, the expressive ability and generalization ability of the network can be improved by learning the feature importance of each channel, so as to obtain better performance and effect.

### 3.4. Loss Function

Unlike existing supervised learning methods that require labels, the proposed model uses unsupervised learning methods that do not require labels, which can help reduce costs. Because our optimization goal is to maximize the SE, the SE can be calculated by Equation (2) after obtaining *N* phase-shift values of the reflecting elements at the output layer. Therefore, the loss function can be defined as a negative value of the SE. Considering that the output of the network needs to be limited to [0,2π], a penalty term is added to the loss function to limit the output to avoid overfitting, thereby enhancing the model’s generalization ability. When the output falls within the range of 0 to 2π, the penalty term is set to 0, indicating that no adjustment is applied to the output. However, when the output exceeds 2π, the penalty term functions by subtracting the excess portion from the output, ensuring that the output satisfies the constraint. The same adjustment applies when the output is below 0. Hence, we define the loss function as

(11) $$\begin{array}{c} Loss=-\frac{1}{B}\sum_{i=1}^{B}\left[\mathrm{SE}\left(\left\{\hat{\theta }\right\}\right)+\lambda R\left(\hat{\theta }\right)\right] \end{array}$$

where *B* is the batch size, λ is the hyperparameter tuning factor and its optimal value can be obtained through experiments, $\left\{\hat{\theta }\right\}$ is the output value from the neural network, and $R(\hat{\theta })$ is the penalty function (tf.clip_by_value is a function in TensorFlow that limits the values in a tensor to a certain range), as follows:

(12) $$\begin{array}{c} R\left(\hat{\theta }\right)=\sum_{j=1}^{N}norm{\left[clip\_by\_value\left(\hat{\theta },0,2\pi \right)-\hat{\theta }\right]}^{2} \end{array}$$

## 4. Simulation Results

### 4.1. Simulation Settings

The simulation results are provided in this section to evaluate the performance of the proposed model. The considered system is assumed to consist of a multi-antenna base station, an IRS, and a multi-antenna user. This article considers a two-dimensional coordinate system where the distance between the base station and the IRS is fixed at 80 m, and the user is placed near the IRS within a region of a 2 m radius with the IRS as the center. Furthermore, it is assumed that all channels experience both large-scale and small-scale fading. The path loss model is adopted for large-scale fading, as follows:

(13) $$\begin{array}{c} \beta \left(d\right)=\beta_{0}{\left(d/d_{0}\right)}^{-\alpha } \end{array}$$

where $\beta_{0}$ is the path loss at the reference distance $d_{0}$ and $d_{0}$ is equal to 1 m, *d* is the actual link distance, and the fading coefficient α ranges from 2 to 4. The fading coefficients between the base station and the user, between the base station and the IRS, and between the IRS and the user are set to 3.5, 2.2, and 2.8, respectively [18]. Taking into account small-scale fading, we assume that all relevant channels adopt the Rician fading channel model. Therefore, the channel **G** between the base station and the IRS is given by the following equation:

(14) $$\begin{array}{c} G=\sqrt{\frac{\kappa_{t}}{\kappa_{t}+1}}G^{LOS}+\sqrt{\frac{1}{\kappa_{t}+1}}G^{NLOS} \end{array}$$

where $\kappa_{t}$ represents the Rician factor. Let $\kappa_{t}$, $\kappa_{r}$, and $\kappa_{d}$ denote the Rician factors of the channels G, $h_{r}$, and $h_{d}$, respectively. $G^{LOS}$ and $G^{NLOS}$ represent the line-of-sight and non-line-of-sight components, respectively, where the non-line-of-sight component is modeled using Rayleigh fading. The channels between the base station and the user, and between the IRS and the user, are also generated through a similar process. As extensively set in [6,18], the user is randomly placed near the IRS. Therefore, both $\kappa_{t}$ and $\kappa_{r}$ are randomly generated. Moreover, due to the large distance and presence of random scattering between the base station and the user, $\kappa_{d}$ is set to 0. In this article, the number of IRSs is set to 40, the bandwidth is set to 10 MHz, and the noise power is set to −80 dBm [18]. The above settings can be used to generate training and testing data.

Our simulation platform is developed by using Python 3 and utilizes the deep learning framework TensorFlow for the construction and training of the ACNet model. In order to fully train and correctly evaluate the model, $8\times 10^{4}$ and $2\times 10^{4}$ data samples are generated as the training set and validation set, respectively. The number of filters in the CNN layer, the size of the convolution kernel, and the number of neurons in the FC layer are shown in Figure 3 and Figure 4. We use the adaptive moment optimizer (ADAM) [42] as the optimizer to update the weights, and the initial learning rate is set to 0.001 and the batch size is set to 2000. To speed up at the beginning of training and reduce oscillations at the end of training, the learning rate decays to 91% of its original value when there is no decrease for 10 consecutive epochs on the validation set. Also, an early stopping mechanism of 50 epochs is set to prevent overfitting to obtain the model with the best validation performance.

### 4.2. Performance Analysis

To verify the performance and generalization of the proposed method, we consider two MIMO systems, 16×4 and 8×4, and compare them with the following benchmark schemes:

- **AO:** The optimal transmit covariance matrix is obtained for the direct channel, and then (P1) is solved by the conventional AO method proposed in [18] with a convergence threshold set to $\epsilon =10^{-4}$.
- **ACNet without attention**: The proposed algorithm without the attention module SENet.
- **LPSNet**: A fully connected neural network-based algorithm, i.e., the solution proposed in [35]. The number of hidden layers is set to 2, and the input data are a one-dimensional vector of length 2MK(N+1).
- **Genetic algorithm**: Iteratively mutates to find the optimal value, and the number of iterations is set to 50.
- **Random phase**: The value of $\theta_{n}$ is randomly selected from the interval [0, 2π].
- **Without IRS**: There is only the direct channel between the base station and the user.

**Figure 5:**
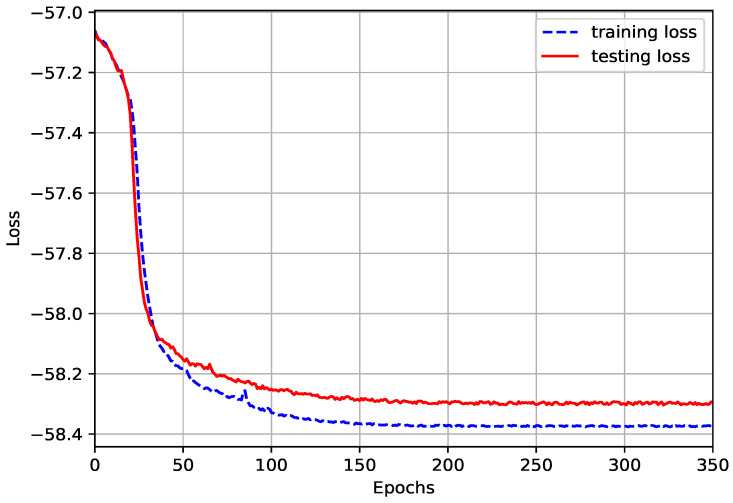
Changes in training error with training times.

The training error of the proposed ACNet model varies with the number of training times as shown in Figure 5. It can be seen that the training error decreases with the increase in the number of training times. When the number of training times reaches about 150, the training error area is stable and converges.

**Figure 6:**
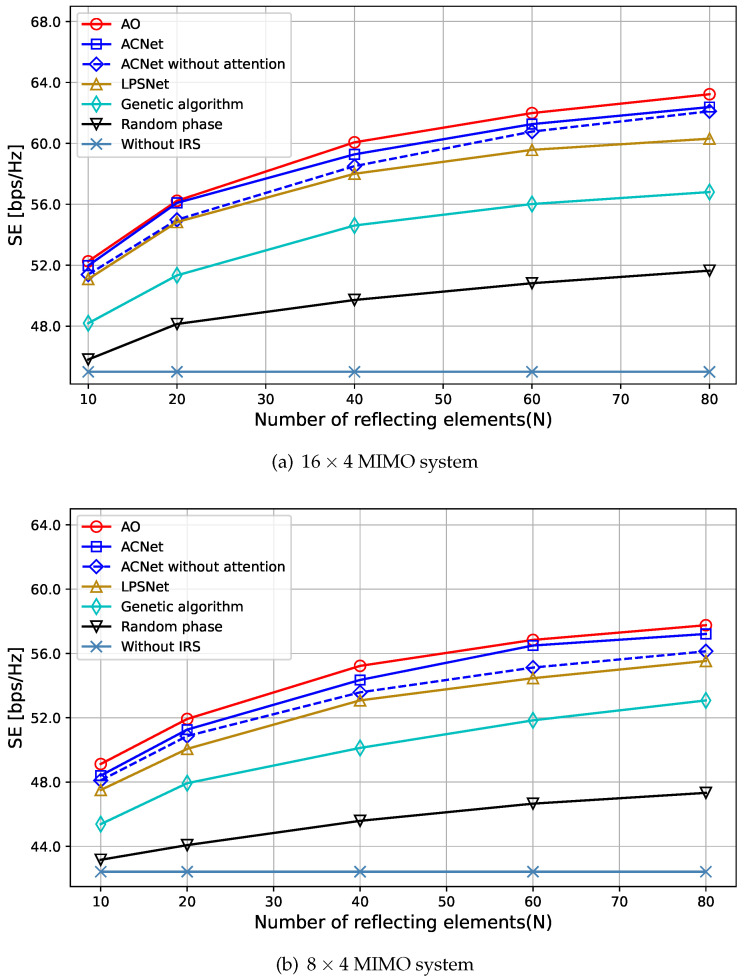
Impact of N ($P_{BS}$ = 40 dBm).

As shown in Figure 6, it illustrates the relationship between the SE and the number of IRS-reflecting elements *N* in the 16×4 and 8×4 MIMO systems when $P_{BS}$ = 40 dBm. From both systems, we can observe that all schemes with an IRS outperform the scheme without an IRS, and the SE of the system increases with the increase in the number of reflecting elements. The reason is that more reflecting elements can enhance the effective gain brought by the reflection path, provide additional spatial degrees of freedom for the system, make passive beamforming more flexible, improve the channel quality of the BS-IRS-UE link, and thus improve the overall SE of the system. It can be found that the proposed algorithm can significantly improve the SE of the system under various numbers of IRS-reflecting elements. In addition, it can be seen from Figure 6a that the proposed algorithm can almost achieve the performance of the baseline algorithm in [18] when *N* = 20, and it can achieve 98.1% of its performance in other cases. Furthermore, the proposed algorithm outperforms the fully connected neural network in [35], and even without the attention module, it still outperforms the algorithm in [35]. This is because the proposed algorithm uses three-channel input data, which have better performance, and the convolutional layer can better extract features from the input data. In addition, the performance of the genetic algorithm is inferior to that of neural network methods and the AO algorithm. This phenomenon arises due to the inherent complexity of finding the optimal value for the non-convex optimization objective presented in this paper. Genetic algorithms, which heavily rely on significant time and computational resources to search for the optimal solution, often struggle to obtain the optimal solution. Furthermore, a genetic algorithm is a mutation-based algorithm and may get trapped in local optima during the search process, failing to find the global optimum.

**Figure 7:**
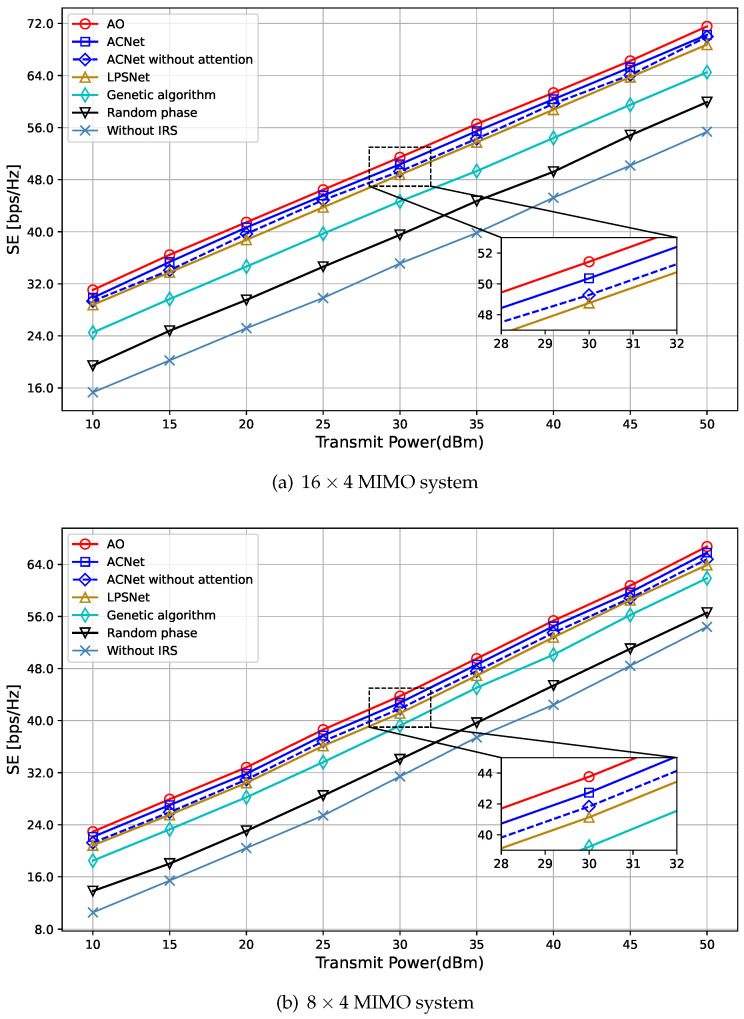
Impact of transmit power (N=40).

As shown in Figure 7, it depicts the relationship between the SE and the BS transmission power $P_{BS}$ in the 16 × 4 and 8 × 4 MIMO systems when *N* = 40. Similar to the results in Figure 6, all schemes with an IRS outperform the scheme without an IRS, and the SE of the system increases with the increase in the BS transmission power. Compared with LPSNet, the genetic algorithm, and the scheme with a random phase shift in [35], the proposed algorithm shows better performance in system performance and can achieve almost the same algorithmic performance as [18]. This is because the proposed scheme is optimized from the input structure to the network model. Firstly, a more effective three-channel input structure is designed for the model rather than simply vectorizing the CSI. It is beneficial for the model to better exploit the features of the data. Secondly, the attention mechanism is added to the neural network, which can make the network pay more attention to important features and information and learn the input data more effectively, thereby improving the performance of the model.

**Figure 8:**
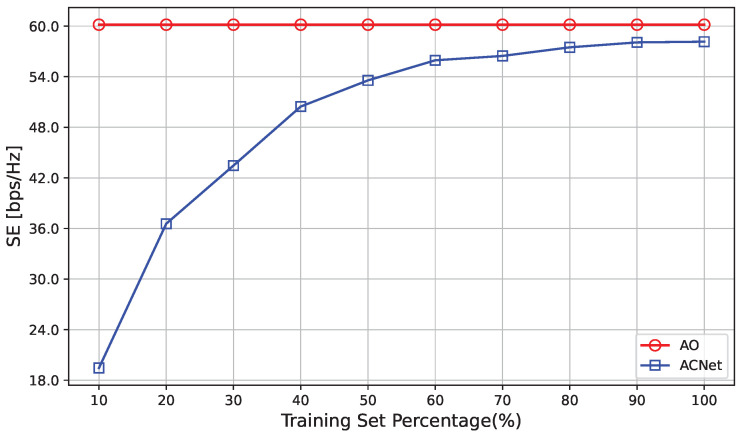
Impact of the number of training set percentage.

It can be observed from Figure 8 that when the training set size increases, the network performance continues to improve and can fully learn the optimal IRS phase-shift configuration. At the same time, when the size of the training set reaches 60% or more, the difference in SE is small, which indicates that the proposed algorithm can effectively estimate the optimal IRS phase shift with a small training set size, thereby maximizing SE and reducing training costs. This result shows that the proposed algorithm is robust and scalable, making it more valuable in practical applications. Because the size of the training set has a small effect on the performance of the algorithm, the training cost can be reduced by reducing the size of the training set.

### 4.3. Computational Complexity

According to [18], the complexity of the traditional AO algorithm is $O(KM\left(N+\min\left(K,M\right)\right)L+\left(\left(3K^{3}+2K^{2}M+M^{2}\right)N+KM\min\left(K,M\right)\right)I)$, where L is the number of initialization phase shifts and I is the number of iterations. Furthermore, according to [35], the computational complexity of LPSNet is $O(\max\left(KMN^{2},LN^{2}\right))$, where L represents the number of hidden layers in LPSNet. After the training of the neural network is completed, the parameters are fixed, which transforms the optimization problem (P1) into a matrix calculation. Therefore, the complexity of the algorithm proposed in this paper is $O(MNK+N^{2}+C^{2}/r)$, which is similar to LPSNet and significantly lower than that of the traditional optimization method.

## 5. Conclusions

In this paper, we proposed an attention-assisted unsupervised learning method to optimize the phase shift of the IRS for improving the SE of IRS-assisted MIMO systems. Specifically, an effective input structure was constructed for the optimization problem, which was used as multi-channel input data for the network. An attention module was inserted into the network to improve the model’s accuracy, and a penalty term was added to the loss function according to constraints to ensure that the output meets the requirements. The simulation results show that the proposed algorithm had great convergence and robustness and can achieve similar performance with lower complexity compared to existing solutions in different conditions. Furthermore, due to the strong learning ability and adaptability of the proposed algorithm, it can be effectively adjusted and optimized in different application scenarios, providing a more comprehensive and flexible solution for IRS system design and optimization. In future work, we can extend the scenario to multi-user settings. In addition, the imperfect CSI, hardware impairment, and advanced IRS technologies such as STAR-IRS can also be considered. 

## Figures and Tables

**Table 1:** Summary of related conventional method literature.

Ref.	System Model	Goal	Approach
[10]	Multi-user MISO	Energy efficiency maximization	Fractional programming, gradient descent, and alternating maximization
[13]	MISO	Achievable rate maximization	AO, SDR
[14]	Multi-user MISO	Weighted sum-rate maximization	SDR
[15]	SISO	Achievable rate maximization	SCA, SDR
[16]	Multi-user MISO	Sum-rate maximization	Combining alternating maximization with majorization–minimization
[18]	MIMO	Channel capacity maximization	AO
[19]	MISO	SE maximization	Fixed-point iteration and manifold methods
[20]	MISO	Total received signal power maximization	SDR, AO
[21]	Multi-user MISO	Weighted sum-rate maximization	Lagrangian manifold and Riemannian manifold techniques

## Data Availability

Not applicable.
